# One-Step Solvothermal Method to Prepare Ag/Cu_2_O Composite With Enhanced Photocatalytic Properties

**DOI:** 10.1186/s11671-016-1246-7

**Published:** 2016-01-19

**Authors:** Xiaolong Deng, Chenggang Wang, E. Zhou, Jinzhao Huang, Minghui Shao, Xianqi Wei, Xiaojing Liu, Meng Ding, Xijin Xu

**Affiliations:** School of Physics and Technology, University of Jinan, 336 Nanxin Zhuang West Road, Jinan, 250022 Shandong Province People’s Republic of China

**Keywords:** Ag/Cu_2_O, Composites, Photocatalytic activity

## Abstract

Ag/Cu_2_O microstructures with diverse morphologies have been successfully synthesized with different initial reagents of silver nitrate (AgNO_3_) by a facile one-step solvothermal method. Their structural and morphological characteristics were carefully investigated by means of X-ray diffraction (XRD), scanning electron microscopy (SEM), and transmission electron microscopy (TEM), and the experimental results showed that the morphologies transformed from microcubes for pure Cu_2_O to microspheres with rough surfaces for Ag/Cu_2_O. The photocatalytic activities were evaluated by measuring the degradation of methyl orange (MO) aqueous solution under visible light irradiation. The photocatalytic efficiencies of MO firstly increased to a maximum and then decreased with the increased amount of AgNO_3_. The experimental results revealed that the photocatalytic activities were significantly influenced by the amount of AgNO_3_ during the preparation process. The possible reasons for the enhanced photocatalytic activities of the as-prepared Ag/Cu_2_O composites were discussed.

## Background

Over the past decades, the environmental problem, especially wastewater induced by organic dye pollutants, has become a fatal issue accompanying the rapid industry growth, which restricted the sustainable development of human beings [1–3]. Therefore, a great effort has been made for seeking the highly active photocatalysts which could be applied for the environmental remediation [4]. Recently, the hybrid structures, such as nanocomposites, which group the various materials with different properties together to offer the potential enhanced functions, have attracted much more attention [5, 6]. Metal-metal oxide semiconductor materials as one type of these hybrid structures have also been widely investigated due to their potential applications in the fields such as sensing [7, 8], antibacterial [5], charge-transfer process [9–11], optoelectronics [12, 13], energy storage [14], and catalysis [15, 16]. Additionally, it is believed that in the metal-semiconductor composites, metal deposits could act as the electron sinks which trap the photoinduced electrons transferring from the conduction band of semiconductor, while the photoinduced holes could remain on the semiconductor surface, and thus, the recombination of photoinduced electron-hole pairs could be prevented resulting in the improvement of photocatalytic efficiency [17, 18]. Among these metal-semiconductor hybrid structures, Ag/Cu_2_O composites have been extensively explored based on the following reasons: (1) Ag, as one kind of relatively cheap noble metal, has also been investigated at nanoscale driven by its excellent sensing properties [19, 20], catalytic activities [21, 22], optical properties [23], thermal properties [24, 25], and inkjet ink particles [26]; (2) Cu_2_O, as a typically low-cost and nontoxic p-type semiconductor, has a narrow direct bandgap of 2.0–2.2 eV, which could be used as photocatalysts under visible light [17, 27].

Cu_2_O has been first investigated as a visible light-driven photocatalyst for water splitting since 1998 [28]. After that, many efforts have been made to improve the photocatalytic efficiency from the two aspects: (1) modulating the growth process to control the chemical stability, size, morphology, and architecture of Cu_2_O [29–34]; (2) hindering the recombination of photogenerated electron-hole pairs [35] and photocorrosion [36, 37]. For Cu_2_O-based photocatalysts, some actions, such as element doping [38–40] and heterojunction forming [41, 42], have been taken to enhance the photocatalytic activity compared with pure Cu_2_O. Moreover, forming composite was also a very important approach to promote the photocatalytic efficiency for Cu_2_O-based material [43, 44]. As mentioned above, Ag/Cu_2_O, as an important composite, has also been considered a way to enhance the photocatalytic activity of Cu_2_O [16–18, 27, 45]. Generally, Ag could be synthesized by various methods to form different morphologies, such as electrolysis method [46], biological method [47], reducing method [16], photocatalytic process [18], soaking method [27], and polyol process [45]. Likewise, there were many approaches to fabricate controllable Cu_2_O structures, including hydrothermal method [45], solvothermal method [40], solution method [6, 16–18], and electrodeposition method [14, 27]. However, there are only a few reports to investigate the effect of Ag content on the photocatalytic efficiency of Ag/Cu_2_O nanocomposites prepared by electron beam irradiation method [48]. So far, little information is available on the Ag content effect for photocatalytic properties of Ag/Cu_2_O synthesized by a facile one-step solvothermal method.

In this work, a series of Ag/Cu_2_O microstructures were fabricated by a facile solvothermal method by adding different amounts of silver nitrate (AgNO_3_). The effect of Ag content on structures and morphologies of the as-synthesized Ag/Cu_2_O composites were systematically investigated. Furthermore, the photocatalytic activities of Ag/Cu_2_O composites prepared with different amounts of AgNO_3_ for methyl orange (MO) dye in aqueous solution were performed. The results revealed that the photocatalytic activities of the as-prepared samples showed the maximal efficiency on degradation of MO related to the suitable amount of AgNO_3_. The possible reasons for enhanced photocatalytic activities of the as-prepared Ag/Cu_2_O composites were proposed.

## Methods

### Synthesis

All the chemical reagents, such as copper (II) nitrate trihydrate (Cu(NO_3_)_2_·3H_2_O), AgNO_3_, ethylene glycol (EG), and MO, purchased from Sinopharm Chemical Reagent Co., Ltd. (SCRC; China), were of analytical grade and used without further purification. Typically, the samples were prepared as follows, similar to the previous report [40, 49]: 4 mmol Cu(NO_3_)_2_·3H_2_O and certain amount of AgNO_3_ were dissolved into 80 mL ethylene glycol followed by vigorous stirring to form a homogeneous solution. The mixture was then transferred into 100 mL Teflon-lined stainless steel autoclave. Thereafter, the sealed autoclave was kept at 140 °C for 10 h, followed by cooling down to room temperature naturally. The as-prepared precipitants were collected by centrifugation and washing with deionized water and ethanol several times. Finally, the products were obtained by drying the precipitants at 60 °C for 12 h in a vacuum oven. The samples were named as CA-0, CA-0.2, CA-0.5, CA-1, and CA-2 for the AgNO_3_ amounts of 0, 0.2, 0.5, 1, and 2 mmol, respectively.

### Characterization

X-ray powder diffraction (XRD) patterns of the as-prepared samples were analyzed by a German X-ray diffractometer (D8-Advance, Bruker AXS, Inc., Madison, WI, USA) equipped with Cu *K*α radiation (*λ* = 0.15406 nm). The morphologies of the as-synthesized products were observed by a field emission scanning electron microscope (FESEM; FEI Quanta FEG250, FEI, Hillsboro, USA) and transmission electron microscopy (TEM; JEOL-200CX, JEOL, Tokyo, Japan). X-ray photo-electron spectroscopy (XPS) was performed on a Thermo ESCALAB 250XI electron spectrometer equipped with Al *Kα* X-ray radiation (*hν* = 1486.6 eV) as the source for excitation. The Brunauer-Emmett-Teller (BET) specific surface areas of the products were investigated by N_2_ adsorption isotherm at 77 K using a specific surface area analyzer (QUADRASORB SI, Quantachrome Instruments, South San Francisco, CA, USA).

### Photocatalytic Test

The photocatalytic activities of the as-prepared Ag/Cu_2_O samples were performed by a UV-vis spectrophotometer (TU-1901, Beijing Purkinje General Instrument Co., Ltd, Beijing, China) at room temperature in air under visible light irradiation, which was similar to the previous reports [40, 49]. The visible light was generated by a 500-W Xe lamp equipped with a cutoff filter to remove the UV part with wavelength below 420 nm. In brief, a suspension was formed by dispersing 30 mg of powder into 50 mL of 20 mg/L MO aqueous solution. After that, the suspension was kept in dark for 30 min with stirring to reach the adsorption-desorption equilibrium of MO on the surface of Ag/Cu_2_O samples. Ca. 3 mL suspension was taken out after a given irradiation time interval and centrifuged to filtrate the sample powders for the following UV-vis spectra test. The concentration of MO was characterized by measuring the absorbance properties at 464 nm in UV-vis spectra to illuminate the photocatalytic activities.

## Results and Discussion

### Structural and Morphological Characterization of Samples

**Fig. 1:**
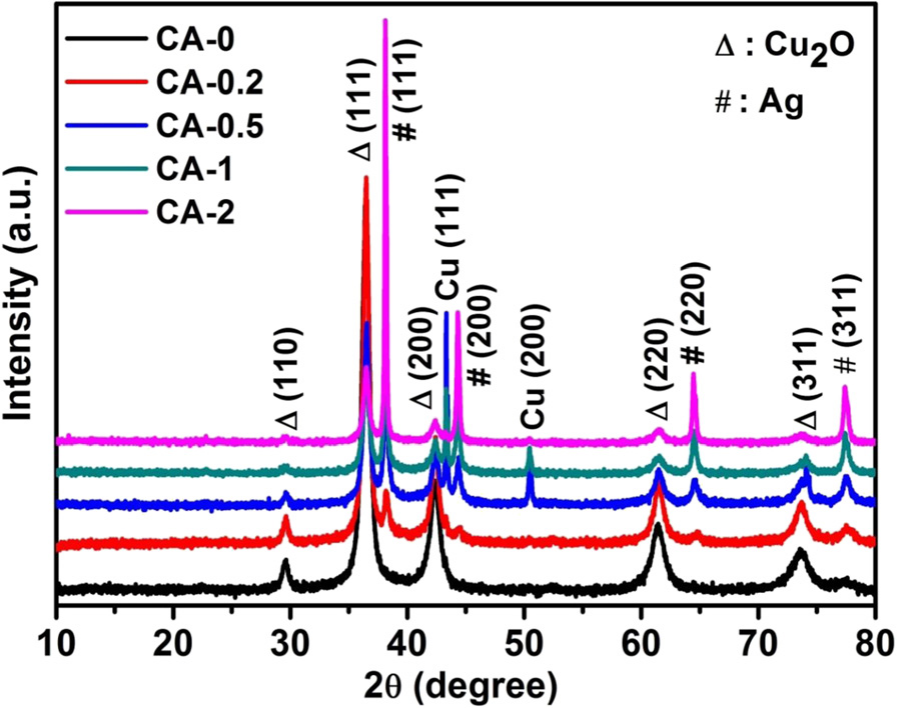
XRD patterns of the as-prepared Ag/Cu_2_O composites with different amounts of AgNO_3_ (CA-0 0 mmol, CA-0.2 0.2 mmol, CA-0.5 0.5 mmol, CA-1 1 mmol, CA-2 2 mmol)

The structural properties of the as-synthesized Ag/Cu_2_O composites are characterized as shown in Fig. 1. The peaks marked with “Δ” and “#” correspond to Cu_2_O and Ag phases, respectively. The XRD pattern of sample CA-0 could be perfectly indexed into cubic Cu_2_O (Joint Committee on Powder Diffraction Standards (JCPDS) no. 78-2076). There are no other characteristic peaks in sample CA-0, which demonstrate the pure phase of CA-0 with a cubic symmetry. However, for other samples (CA-0.2, CA-0.5, CA-1, and CA-2), the diffraction peaks illustrate the existence of both Cu_2_O and Ag in Fig. 1. Furthermore, Cu phases (JCPDS no. 85-1326) are presented in the samples CA-0.5 and CA-1. It is found that the intensity of the diffraction peaks corresponding to Ag phases are enhanced, while that of Cu_2_O phases are inhibited with the increase of AgNO_3_ amounts, which confirm the dominant phase in the Ag/Cu_2_O composites’ change from Cu_2_O to Ag.

**Fig. 2:**
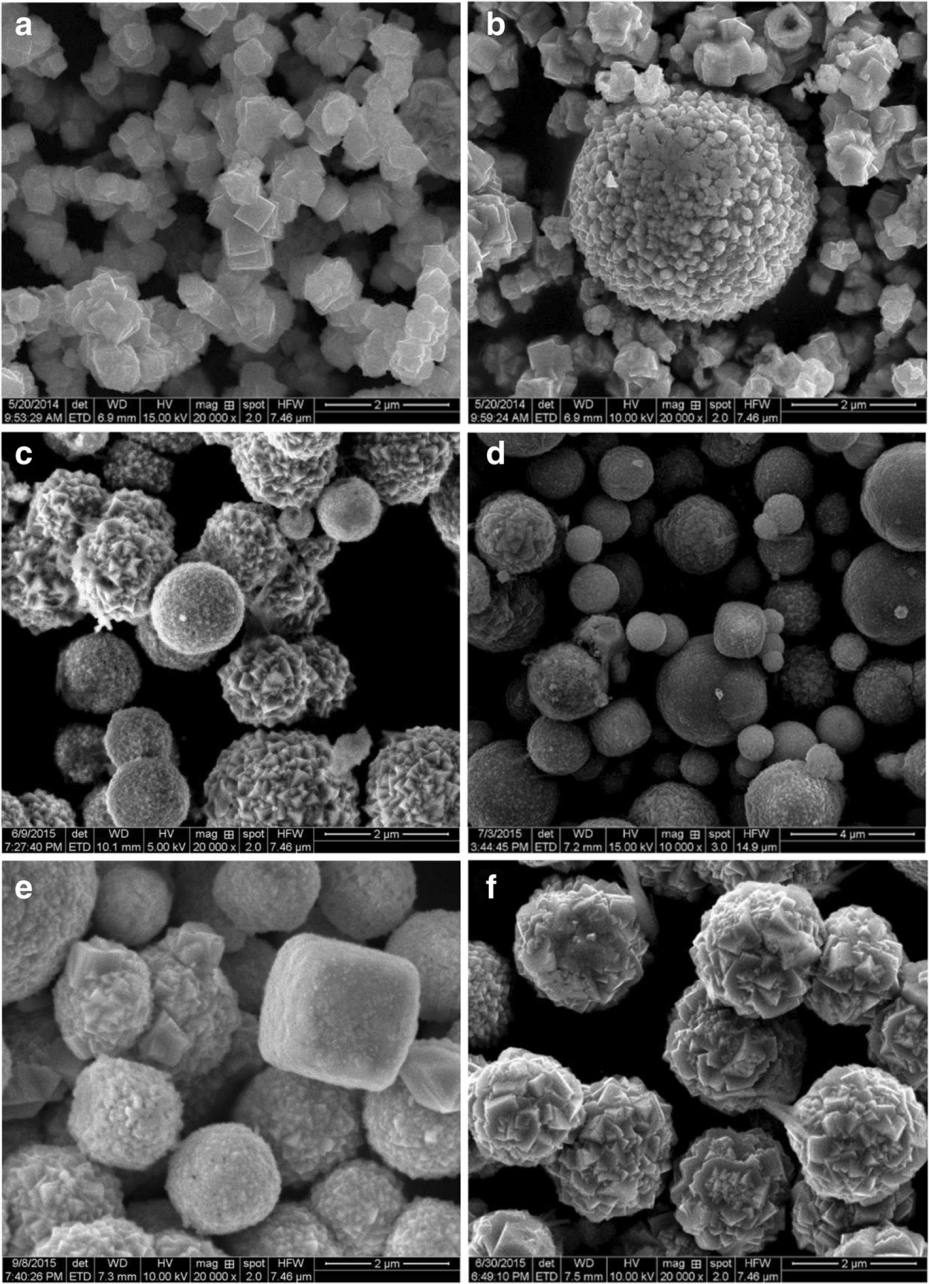
SEM images of the as-grown samples with different amounts of AgNO_3_ during the preparing process: **a**, **b** CA-0, **c** CA-0.2, **d** CA-0.5, **e** CA-1, and **f** CA-2

**Fig. 3:**
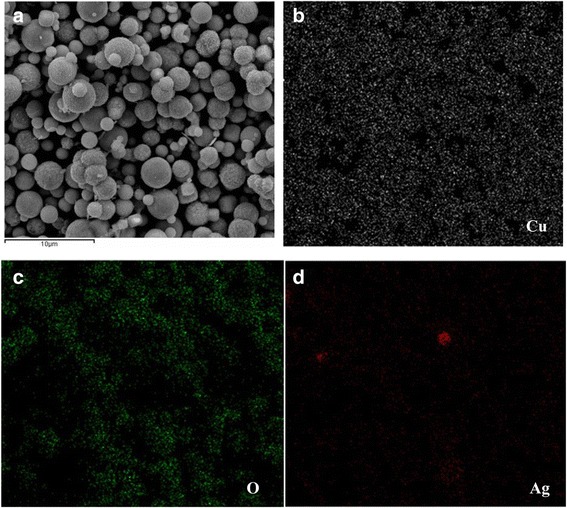
Typically elemental mapping images of CA-0.5 sample. **a** The corresponding SEM image of the mapping area, **b** Cu mapping, **c** O mapping, and **d** Ag mapping

The morphologies with different amounts of AgNO_3_ are shown in Fig. 2. For CA-0, the hierarchical structure was mostly composed of regular cubic particles with size of about 500 nm, as shown in Fig. 2a, as well as a few spherical particles, as shown in Fig. 2b. Once AgNO_3_ was added in the preparing process, the morphologies of the final products were almost entirely transformed into spheres with relatively smooth surface for CA-0.2 sample (Fig. 2c), besides some rough spheres consisting of pyramid particles. Figure 2d displays the as-grown sample CA-0.5 which was composed of nonuniform spheres with the diameter of 500–3500 nm, as well as some irregular structures. For samples CA-1 and CA-2, the SEM images (Fig. 2e, f) show the rough surface of spheres with relatively homogeneous diameter dispersion, while some cubic structures were also observed in sample CA-1. Typically, Fig. 3a shows the large-area (several tens micrometers) SEM image of the as-prepared CA-0.5 sample with spherical structures, and the corresponding energy dispersive X-ray spectroscopy (EDS) elemental mappings in Fig. 3b–c shows that the distributions of Cu, O, and Ag elements shown in Fig. 3d are approximately homogeneous even in the large area.

**Fig. 4:**
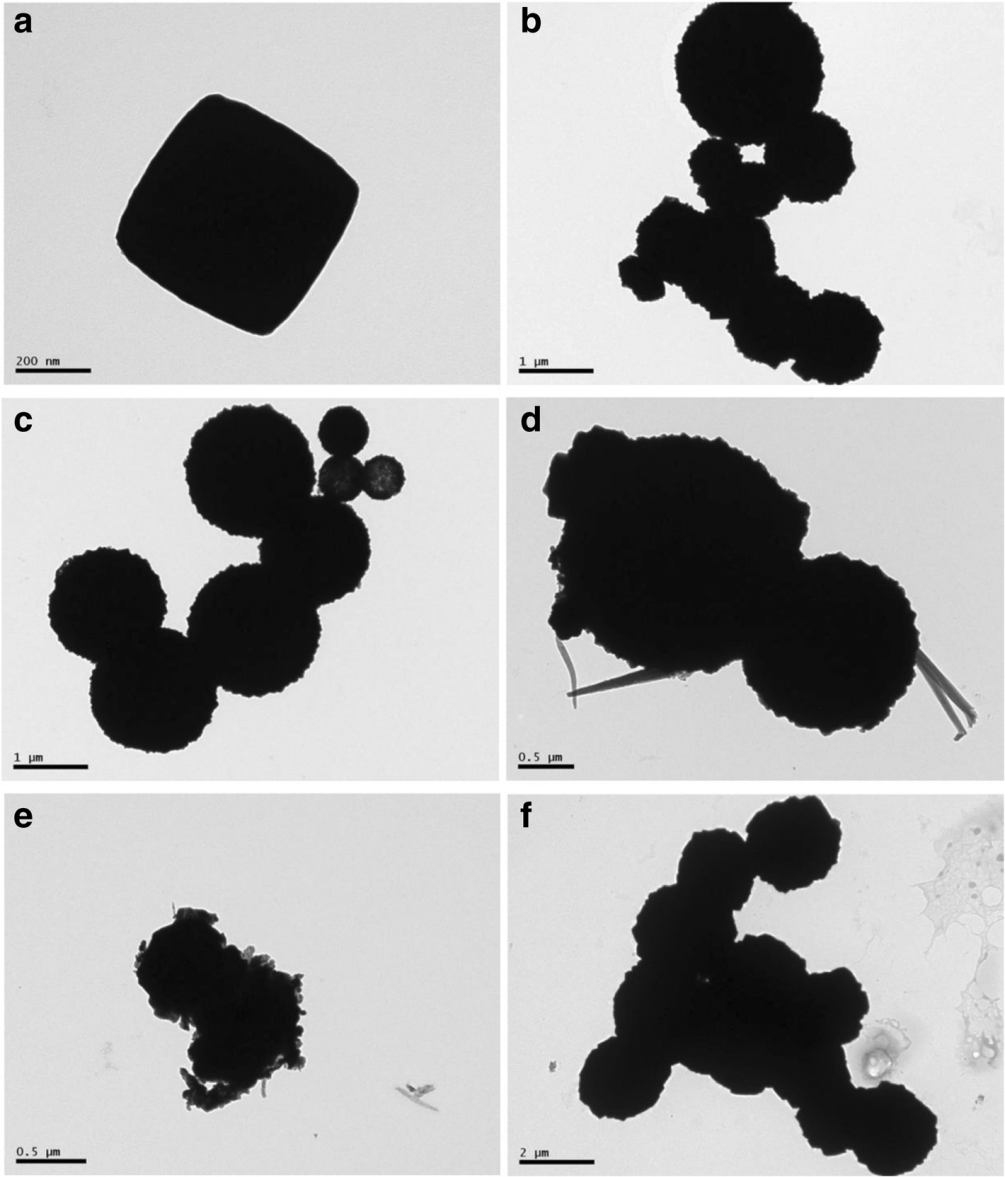
Typical TEM images of the as-prepared Ag/Cu_2_O samples. **a**, **b** CA-0; **c** CA-0.2; **d**, **e** CA-0.5; and **f** CA-2

TEM observations as shown in Fig. 4 for the as-prepared Ag/Cu_2_O samples further corroborate the observed morphologies with SEM. TEM images of sample CA-0 (Fig. 4a, b) depict cubic and spherical particles with size of about 520 nm and 1–2 μm, respectively, consistent with SEM observation. The spheres with homogeneous size distribution of about 1.8 μm are also observed in Fig. 4c for sample CA-0.2. Meanwhile, there are some hollow spheres with the diameter of 550 nm, agreeing with the SEM image. The spheres with heterogeneous diameter and irregular structures for CA-0.5 (Fig. 4d, e) are exhibited, as well as the spheres with uniform diameter and regular structures for CA-2 (Fig. 4f) confirmed the results of SEM observations.

**Fig. 5:**
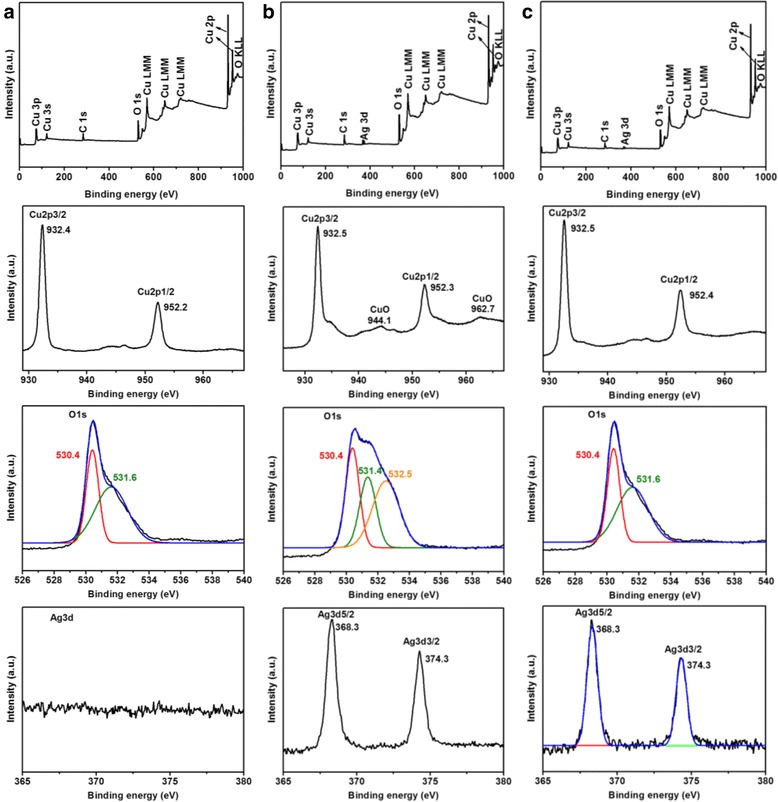
Typical XPS spectrum of the as-grown Ag/Cu_2_O samples including XPS full spectrum, Cu 2p spectrum, O 1s spectrum, and Ag 3d spectrum, respectively. **a** CA-0, **b** CA-0.5, and **c** CA-2

The XPS spectra of the as-prepared samples CA-0, CA-0.5, and CA-2 are depicted in Fig. 5 to further confirm the composition and the elemental states in Ag/Cu_2_O composites. The binding energies are calibrated by C 1s (284.8 eV). All the detected peaks on the survey scan spectra can be ascribed to C, O, and Cu elements for all samples, and Ag peaks are only found in Fig. 5b, c, which is consistent with the previous reports [17, 50]. No other elements’ peaks were observed. C peaks mainly resulted from the hydrocarbon from the XPS instrument itself [50]. From the high-resolution XPS spectrum in the Cu 2p_3/2_ and Cu 2p_1/2_ binding energy region, the two main peaks locating at about 932.5 and 952.3 eV were in agreement with the reported value of Cu_2_O [17, 40, 50, 51], respectively, which confirms the main composition of the structure is Cu_2_O. In addition, the peaks locating at 944.1 and 962.7 eV in the Cu 2p region for CA-0.5 in Fig. 5b may be attributed to CuO due to the open 3d^9^ shell of Cu^2+^ [17, 48, 51]. The O 1s region shown in Fig. 5 could be fit into two main peaks locating at 530.4 and 531.6 eV (or 531.4 eV), which were ascribed to lattice oxygen of Cu_2_O and surface-absorbed oxygen species [17, 40, 48, 50]. Furthermore, a peak at 532.5 eV was observed in the O 1s region shown in Fig. 5b, which originated from the lattice oxygen of CuO [48]. Combining the peaks at 944.1 and 962.7 eV in the Cu 2p region with 532.5 eV in the O 1s region shown in Fig. 5b, it suggests that the existence of thin layer CuO forms on the surface of the sample (CA-0.5) [17]. The Ag 3d XPS spectra show no peaks for sample CA-0 as shown in Fig. 5a, which demonstrates the absence of Ag. However, there are two peaks locating at 368.3 and 374.3 eV shown in Fig. 5b, c in the Ag 3d region, which could be attributed to Ag 3d_5/2_ and Ag 3d_3/2_, respectively, matching well with the reported values of metallic Ag, indicating the existence of Ag with metallic nature [5, 48, 50]. No CuO could be detected from the XRD pattern, which indicates that the trace amount of CuO is present only on the surface of Ag/Cu_2_O composites for sample CA-0.5 [17, 48].

Based on the above results, the synthesis mechanism for Ag/Cu_2_O composites could be proposed as the following, which is similar to the previous reports [5, 27, 40, 49, 50]. The possible chemical reactions should be as follows:

1 $$ {\mathrm{OHCH}}_2{\mathrm{CH}}_2\mathrm{O}\mathrm{H}\to {\mathrm{CH}}_3\mathrm{C}\mathrm{H}\mathrm{O} + {\mathrm{H}}_2\mathrm{O} $$

2 $$ \mathrm{O}\mathrm{H}-{\mathrm{C}\mathrm{H}}_2-{\mathrm{C}\mathrm{H}}_2-\mathrm{O}\mathrm{H}+{\mathrm{C}\mathrm{u}}^{2+}\to {\left[\mathrm{C}\mathrm{u}\left({\mathrm{C}}_2{\mathrm{H}}_6\mathrm{O}\right)\right]}^{2+} $$

3 $$ {\left[\mathrm{C}\mathrm{u}\left({\mathrm{C}}_2{\mathrm{H}}_6\mathrm{O}\right)\right]}^{2+}+{\mathrm{H}}_2\mathrm{O}\to \mathrm{C}\mathrm{u}{\left(\mathrm{O}\mathrm{H}\right)}_2+{\mathrm{H}\mathrm{OCH}}_2{\mathrm{C}\mathrm{H}}_2\mathrm{O}\mathrm{H}+2{\mathrm{H}}^{+} $$

4 $$ 2\mathrm{C}\mathrm{u}{\left(\mathrm{O}\mathrm{H}\right)}_2+2{\mathrm{CH}}_3\mathrm{C}\mathrm{H}\mathrm{O}\to {\mathrm{Cu}}_2\mathrm{O}+{\mathrm{CH}}_3-\mathrm{C}\mathrm{O}-\mathrm{C}\mathrm{O}-{\mathrm{CH}}_3+3{\mathrm{H}}_2\mathrm{O} $$

5 $$ \mathrm{C}\mathrm{u}{\left(\mathrm{O}\mathrm{H}\right)}_2+{\mathrm{CH}}_3\mathrm{C}\mathrm{H}\mathrm{O}\to \mathrm{C}\mathrm{u}+{\mathrm{CH}}_3\mathrm{C}\mathrm{O}\mathrm{O}\mathrm{H}+{\mathrm{H}}_2\mathrm{O} $$

6 $$ 2\mathrm{C}\mathrm{u}+4{\mathrm{CH}}_3\mathrm{CO}\mathrm{O}\mathrm{H}+{\mathrm{O}}_2\to 2\mathrm{C}\mathrm{u}{\left({\mathrm{CH}}_3\mathrm{C}\mathrm{O}\mathrm{O}\right)}_2+2{\mathrm{H}}_2\mathrm{O} $$

The reactions (Eqs. 5 and 6) sufficiently occurred in this work, and finally only Cu_2_O existed in the products due to long reaction time [40, 49]. For sample CA-0, there is no addition of AgNO_3_ resulting in the final product of Cu_2_O. Once AgNO_3_ was added into the solution, the following chemical reactions could occur according to the previous reports [27, 50, 52–54]:

7 $$ 2{\mathrm{Ag}}^{+}+2{\mathrm{CH}}_3\mathrm{C}\mathrm{H}\mathrm{O}\to 2\mathrm{A}\mathrm{g}+{\mathrm{CH}}_3\mathrm{C}\mathrm{O}-{\mathrm{COCH}}_3+2{\mathrm{H}}^{+} $$

8 $$ 2{\mathrm{Ag}}^{+}+{\mathrm{Cu}}_2\mathrm{O}+2{\mathrm{H}}^{+}\to 2{\mathrm{Cu}}^{2+}+2\mathrm{A}\mathrm{g}+{\mathrm{H}}_2\mathrm{O} $$

9 $$ 2{\mathrm{Ag}}^{+}+{\mathrm{Cu}}_2\mathrm{O}+{\mathrm{H}}_2\mathrm{O}\to 2{\mathrm{Cu}}^{2+}+2\mathrm{A}\mathrm{g}+2{\mathrm{OH}}^{-} $$

10 $$ {\mathrm{Ag}}^{+}+\mathrm{C}\mathrm{u}\to {\mathrm{Cu}}^{2+}+\mathrm{A}\mathrm{g} $$

Therefore, the formed Cu^2+^ species may be absorbed on the surface of Cu_2_O to form CuO for some samples such as CA-0.5 [27]. Meanwhile, the formation of Ag covered on Cu_2_O to prevent the further reaction to some extent [27]. Nevertheless, metallic Cu could also be observed in some samples depending on the amount of AgNO_3_. The reason was ascribed to be the broken equilibrium because of the additional reaction of Eq. 10 occurring [54]. When the amount of AgNO_3_ (CA-0.2) was little, the reaction system was no significant difference from that of preparing sample CA-0. Therefore, almost no metallic Cu was observed in this sample (CA-0.2). Once the amount of AgNO_3_ was sufficiently enough (CA-2), there was also almost no existence of Cu due to the complete dissolution of Cu into Cu^2+^ according to Eq. 10. However, if the amount of AgNO_3_ was not enough (CA-0.5 and CA-1), the reaction system equilibrium would be broken compared with the absence of AgNO_3_ (CA-0), and Cu would be partly dissolved resulting in the residual of Cu. Thus, the Ag/Cu_2_O composites were obtained when AgNO_3_ was added during the fabrication process, which was confirmed by XRD patterns and XPS spectra.

### Photocatalytic Activity of Samples

**Fig. 6:**
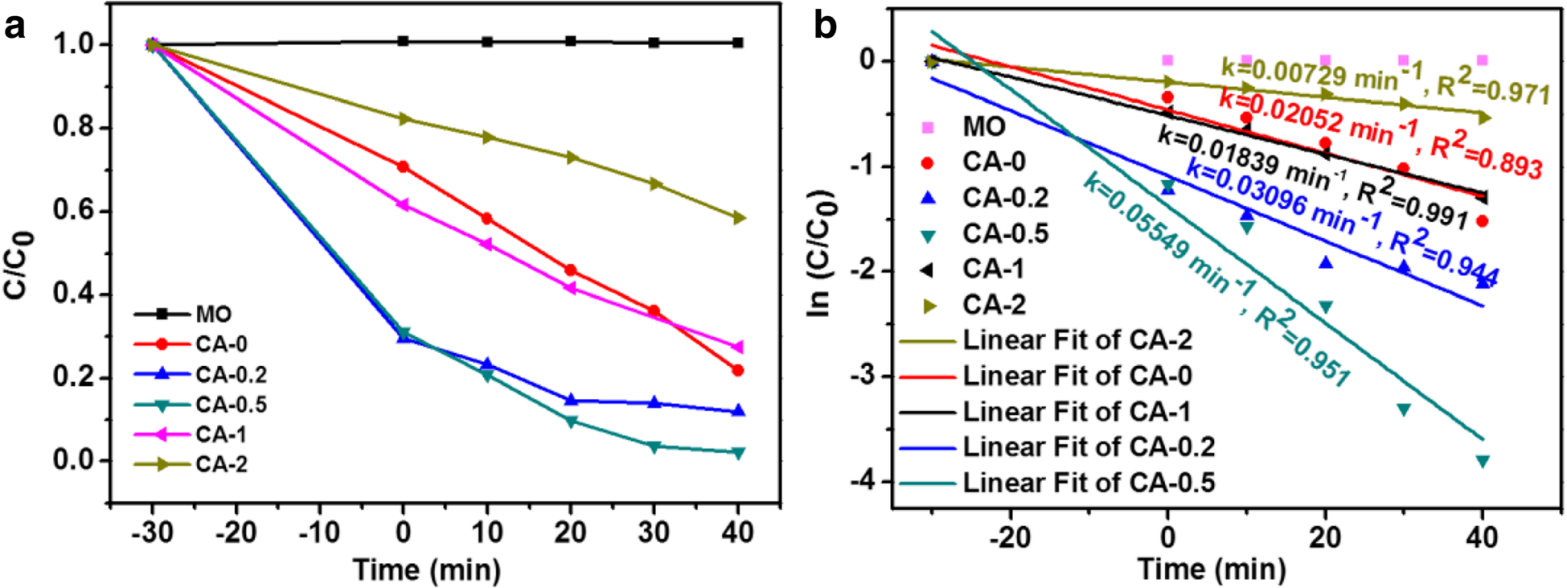
**a** Relative concentration changes of MO as a function of irradiation time for Ag/Cu_2_O samples under visible light irradiation. **b** Photocatalytic degradation kinetics of MO aqueous solution in the presence of Ag/Cu_2_O samples

**Fig. 7:**
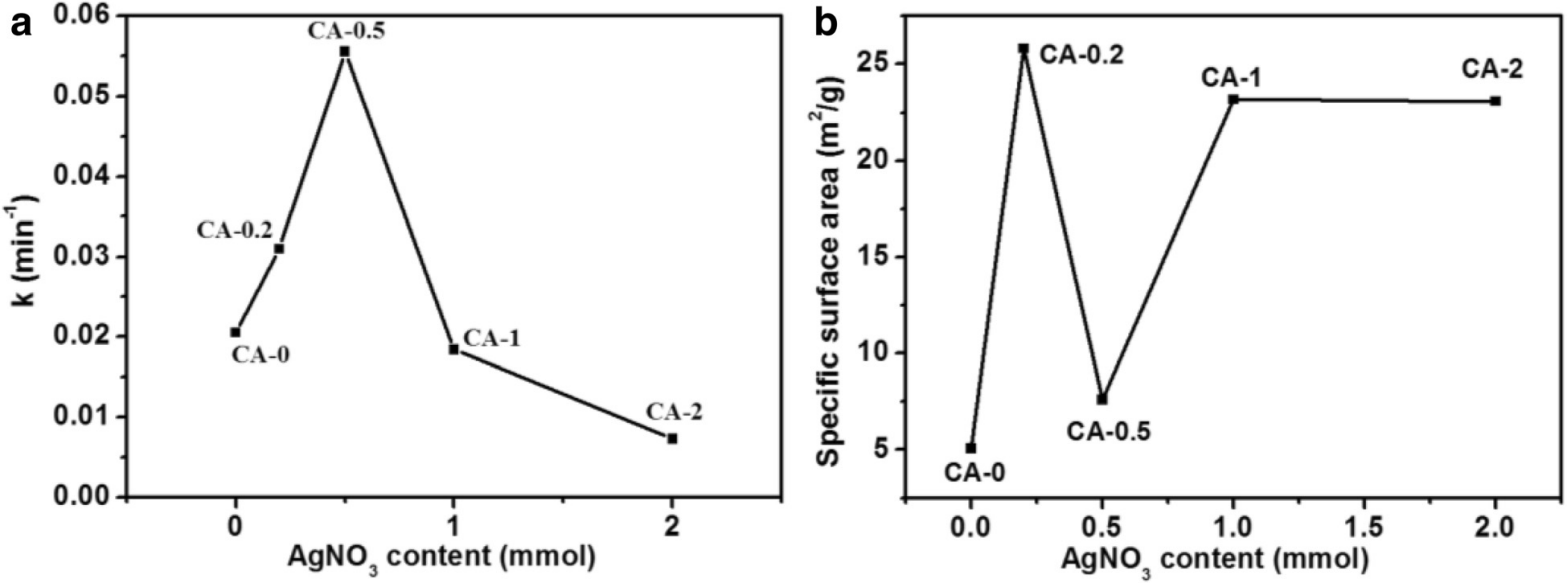
Plots of **a** reaction rate constant and **b** specific surface area versus AgNO_3_ content during the preparing process for Ag/Cu_2_O samples

The photocatalytic activities of the as-prepared samples as photocatalysts on the degradation of MO were evaluated under visible light irradiation, as shown in Fig. 6. From Fig. 6a, it could be seen that the degradation efficiency of Ag/Cu_2_O composite is higher than that of pure Cu_2_O (CA-0) with the increment of AgNO_3_ (CA-0.2 and CA-0.5). However, further increase of AgNO_3_ amount resulted in the decrease of degradation efficiency (CA-1 and CA-2). For a detailed analysis of photocatalytic degradation kinetics of MO aqueous solution, the pseudo first-order model was applied to determine the rate constant of photodegradation with respect to the degradation time when the initial concentration of the pollutant is low, as expressed by Eq. 11 [3, 40, 49, 50].

11 $$ \ln \left(\raisebox{1ex}{$C$}\!\left/ \!\raisebox{-1ex}{${C}_0$}\right.\right)=-kt $$

where *C*_0_ is the initial concentration of MO, *C* is the concentration at time *t*, and *k* is the reaction rate constant. The photocatalytic degradation kinetics of MO aqueous solution on the basis of the plots of ln(*C/C*_0_) versus time *t* was described in Fig. 6b. The rate constant (*k*) was given by the slopes of linear fit and estimated to be 0.02052, 0.03096, 0.05549, 0.01839, and 0.00729 min^−1^ for samples CA-0, CA-0.2, CA-0.5, CA-1, and CA-2, respectively. The rate constant values illustrated that the degradation rates for MO dye with the sequence of CA-0.5>CA-0.2>CA-0>CA-1>CA-2. For intuitive description of rate constant, the values were plotted versus the AgNO_3_ content during the preparing process for Ag/Cu_2_O samples, as illustrated in Fig. 7a. It showed more clearly that the rate constant achieved the maximum at the AgNO_3_ content of 0.5 mmol (sample CA-0.5).

The causes for the vibration of photodegradation rates could be ascribed to as the following according to the literatures [17, 18, 27, 48, 50]: (1) surface plasmon resonance, which means that the photoexcited plasmonic energy in the Ag particles transferred into Cu_2_O resulting in the more generation of electron-hole pairs in the Cu_2_O, which is beneficial to improve the photocatalytic activity; (2) Ag particles act as electron sinks, which means the photogenerated electrons transferring from the conduction band of Cu_2_O to Ag particles, leading to the improvement in photocatalytic activity over an extended wavelength range and to prevent the recombination of photogenerated electron-hole pairs and enhance the interfacial charge-transfer and thus promotes the photocatalytic activity; (3) the enlarged specific surface area also improves the photocatalytic activity by increasing the contact area. However, with the increasement of the AgNO_3_ content, the photocatalytic effect decreases. The specific surface areas of the as-prepared samples are described in Fig. 7b. The trend of specific surface area vibration was not consistent with the photodegradation rate, especially, for CA-0.5. The reason may be ascribed to the morphology transformation due to the addition of AgNO_3_, which was in agreement with the SEM and TEM observations. The photodegradation activity vibration could be explained as follows: the excessive Ag causes the aggregation of Ag particles, resulting in the decrease of capturing the photogenerated electrons and the shield of the visible light absorption by Cu_2_O, leading to the deterioration of photo-utilizing efficiency. In addition to the aforementioned reasons, the morphology was also one of the key factors for the photocatalytic activity. In this work, the morphology transformation experienced the following procedure: regular cube to smooth sphere to rough sphere with the increase of AgNO_3_. The morphology would not only affect the specific surface area of the as-prepared samples as mentioned above but also influence the exposed crystal surfaces as previously reported [55, 56] which had significant impact on the photocatalytic activity. It is reported that [111] surfaces had much higher photocatalytic activity for Cu_2_O and the cubic and spherical particles were mainly covered by [100] or [110] surfaces [55]. Combining SEM and TEM observations, the samples were reasonable to have the sequence of photocatalytic activities as shown in Fig. 7a (CA-0 (cube), CA-0.2 (spheres consisted of pyramid particles), CA-0.5 (spheres composed of pyramid particles and other irregular structures), CA-1 (cube and cube formed sphere), CA-2 (sphere composed of cubic particles)). Finally, Cu contained in some samples (CA-0.5 and CA-1) also contributed to the enhanced photocatalytic activities by promoting the rapid separation of photogenerated electrons and holes in the interfaces between Cu and Cu_2_O [49, 57, 58]. However, the existence of Cu was not the dominant factor responsible for the enhanced photocatalytic activities by comparing the photodegradation rate of CA-1 with CA-0. In a word, the photocatalytic activity of Cu_2_O on decomposition of MO dye in aqueous solution is enhanced by the formation of Ag particles with suitable amount of Ag content, which plays the dominant role, and the morphology effect.

**Fig. 8:**
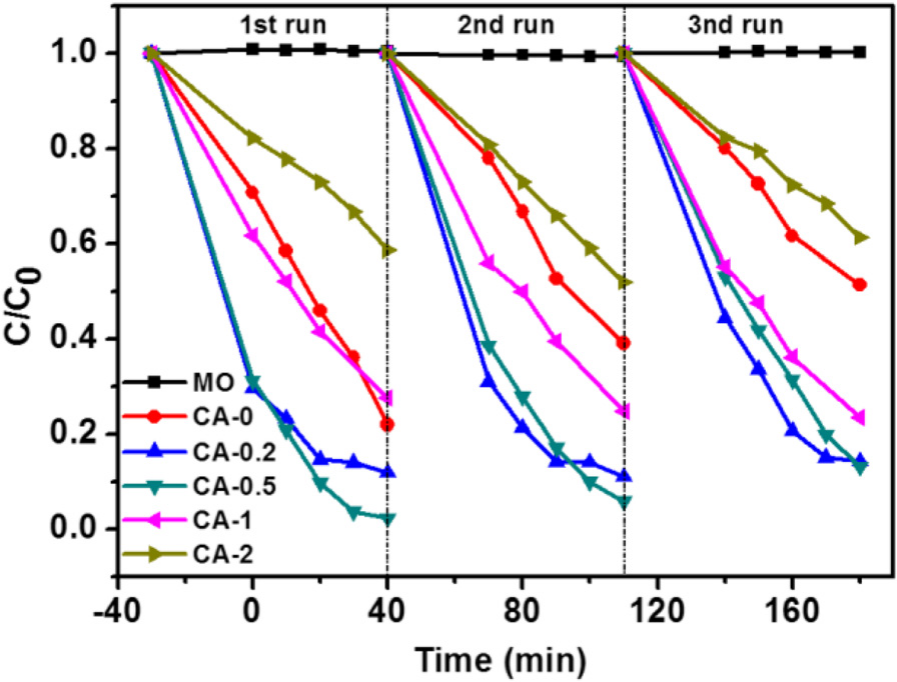
Cycling runs in the photocatalytic degradation of MO aqueous solution in the presence of Ag/Cu_2_O catalysts under visible light irradiation

The cycle runs in the photocatalytic degradation of MO aqueous solution in the presence of Ag/Cu_2_O catalysts under visible light irradiation were investigated to evaluate the durability of Ag/Cu_2_O composite for water treatment. All the experiments were carried out in the same conditions. Figure 8 presents the corresponding results. As observed, the MO degradation ratio has no significant difference after 3 cycles, indicating that the as-prepared Ag/Cu_2_O composites exhibit good durability as photocatalysts for photodegradation of MO in aqueous solution.

**Fig. 9:**
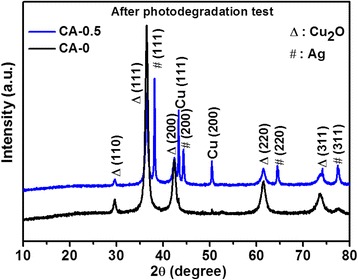
XRD patterns of Ag/Cu_2_O composites (CA-0 0 mmol and CA-0.5 0.5 mmol) after photodegradation measurement

**Fig. 10:**
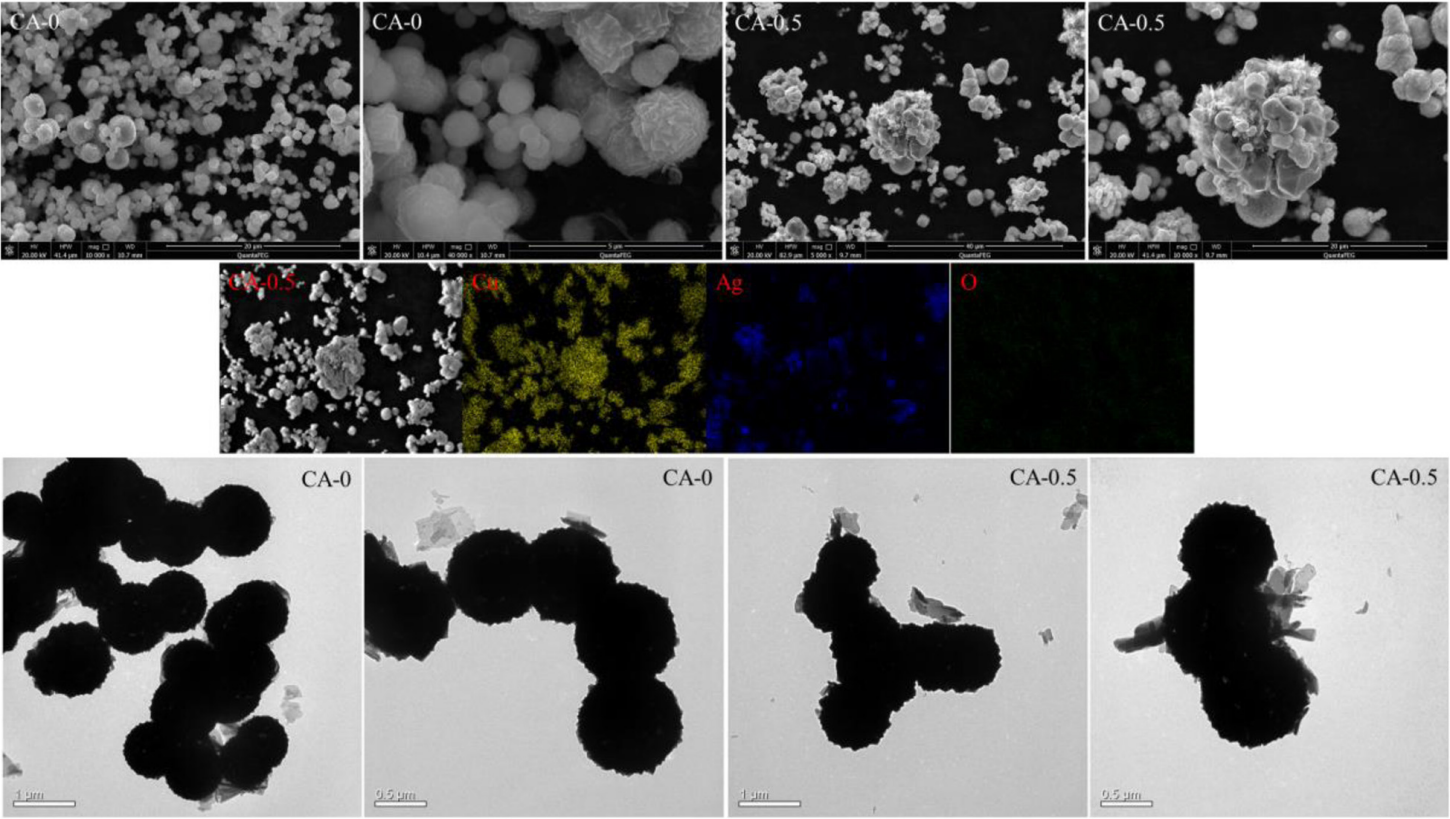
SEM (*upper panel*), elemental mapping (*middle panel*), and TEM (*lower panel*) images of Ag/Cu_2_O composites (CA-0 and CA-0.5) after photodegradation measurement

**Fig. 11:**
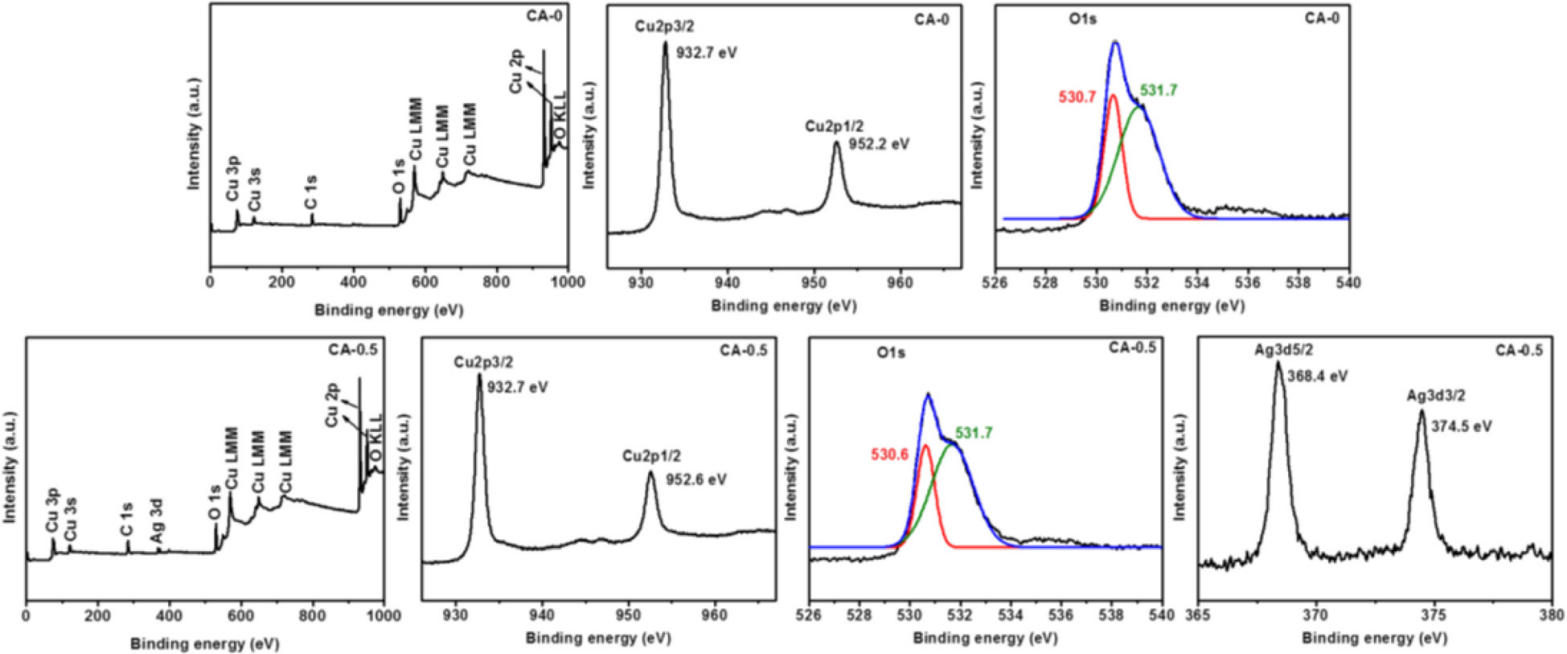
The XPS spectrum of Ag/Cu_2_O composites after photodegradation test including XPS full spectrum, Cu 2p spectrum, O 1s spectrum, and Ag 3d spectrum, respectively. (*Upper panel*) CA-0 and (*lower panel*) CA-0.5

The stability of Ag/Cu_2_O composite on the photodegradation of MO aqueous solution was estimated by characterizing the samples after photocatalytic test. The XRD patterns of CA-0 and CA-0.5 after photodegradation was plotted as shown in Fig. 9, which demonstrated no difference from the initial states depicted in Fig. 1. The morphologies observed by SEM and TEM in Fig. 10 were almost no change compared with the corresponding samples before photodegradation measurement shown in Fig. 2. However, XPS spectra of CA-0.5 after photodegradation were different from the initial state while the spectra of CA-0 kept the same, as depicted in Fig. 11. The peaks denoted CuO in the XPS spectra of CA-0.5 disappeared in the Cu 2p and O 1s regions, which could be ascribed to the reduction of CuO to form Cu_2_O induced by the photogenerated electrons [59]. Nevertheless, the XPS showed no change of the main component of Ag/Cu_2_O composite for the sample CA-0.5 after photodegradation. Therefore, these results confirmed the stability of the as-prepared Ag/Cu_2_O composites for the degradation of MO in aqueous solution under visible light irradiation.

## Conclusions

In summary, Ag/Cu_2_O composites were successfully synthesized by a facile one-step solvothermal method. The structural and morphological properties were characterized by XRD, SEM, TEM, and XPS, which demonstrated that the AgNO_3_ amount during the fabrication process significantly affected the surface, size distribution, morphology, and specific surface area of the as-grown Ag/Cu_2_O composites. The photocatalytic activities of the as-prepared Ag/Cu_2_O composites on the photodegradation of MO dye were evaluated under visible light irradiation. The results illustrated that Ag particles played an important role in the photodegradation of MO by surface plasmon resonance and acting as electron sinks. However, excessive Ag would decrease the photocatalytic activity because of shielding the visible light absorption by Cu_2_O and lowering the capture of photogenerated electrons. The photodegradation of MO was also affected by the morphology of the as-prepared samples, though Ag particles were the dominant factor in this work. The as-prepared Ag/Cu_2_O composites have good stabilities as photocatalysts for photodegradation of MO in aqueous solution, illustrating to be promising in wastewater treatment.
